# Relapse predictors and brain atrophy in antibody-positive and antibody-negative autoimmune encephalitis: a multicenter cohort study

**DOI:** 10.3389/fimmu.2026.1879021

**Published:** 2026-08-25

**Authors:** Xiao-hong Tang, Jing-xian Wang, Hao Li, Kai-xin Zhu, Liao Wu, Lin-kang Bai, Jing-hua Chen, Zhang-xu Lian, Wei Wang, Bin Liu, Teng-fei Liu, Chun-mei Wen, Nuan Wang, Sen Qun, Qing-wei Lai

**Affiliations:** 1 Department of Neurology, Affiliated Hospital of Xuzhou Medical University, Xuzhou, China; 2 Department of Neurology, People’s Hospital of Hongze District, Huaian, China; 3 Department of Medical Records, Nanjing Brain Hospital, Nanjing, China; 4 Department of Neurology, The Fourth Affiliated Hospital of Soochow University, Suzhou, China; 5 Department of Neurology, The First Affiliated Hospital of University of Science and Technology of China, Hefei, China; 6 Department of Neurology, The Affiliated Huaian No.1 People′s Hospital of Nanjing Medical University, Huaian, China; 7 Department of Neurology, The First People’s Hospital of Sihong County, Suqian, China; 8 Department of Oncology, The First Affiliated Hospital of Shandong First Medical University, Jinan, China; 9 Department of Neurology, Xuzhou Central Hospital, Xuzhou, China; 10 Department of Neurology, Yancheng First People’s Hospital, Yancheng, China; 11 Department of Geriatric Medicine, Nanjing Brain Hospital, Nanjing, China; 12 Department of Neurology, Xuzhou Mining Group General Hospital, Xuzhou, China; 13 Department of Neurology, Yancheng Third People’s Hospital, Yancheng, China; 14 Department of Neurology, First People’s Hospital of Xuzhou, Xuzhou, China; 15 Division of Life Sciences and Medicine, University of Science and Technology of China, Hefei, China; 16 Department of Neurology, The First Clinical College, Xuzhou Medical University, Xuzhou, China

**Keywords:** autoimmune encephalitis, brain atrophy, immunotherapy, neuroimaging, relapse

## Abstract

**Background:**

Autoimmune encephalitis (AE) is clinically heterogeneous, but whether relapse predictors and structural correlates of disability differ by antibody status remains unclear. We aimed to identify serostatus-specific predictors of relapse and determine whether brain atrophy is associated with 12-month functional outcome across the AE spectrum.

**Methods:**

In an 11-center retrospective cohort study conducted from 2016 to 2024, we included patients fulfilling 2016 AE diagnostic criteria with ≥12 months of follow-up. Serostatus, relapse, 12-month modified Rankin Scale (mRS), and MRI-defined diffuse cortical atrophy (DCA), medial temporal atrophy (mTA), and cerebellar atrophy were analyzed. Multivariable logistic regression was used to identify relapse predictors within serostatus strata. Functional outcomes and regional brain atrophy patterns were compared between antibody-positive and antibody-negative cohorts.

**Results:**

Among 467 patients (361 antibody-positive, 106 antibody-negative), 1-year relapse rates were 25.5% and 22.6%, respectively. In antibody-positive AE, faciobrachial dystonic seizures were independently associated with relapse (OR = 2.50, *P* = 0.022). Within LGI1-AE, older age at onset was associated with relapse (OR = 1.04, *P* = 0.042). In antibody-negative AE, frequent daily seizures independently predicted relapse (OR = 4.12, *P* = 0.023). Antibody-negative patients had more frequent DCA than antibody-positive patients (32.1% vs 16.6%, *P* < 0.001). Across serostatus groups, moderate-to-severe DCA and mTA was associated with worse 12-month functional outcome (all *P* < 0.05).

**Conclusions:**

Relapse-associated factors differed between antibody-positive and antibody-negative AE. In both cohorts, moderate-to-severe DCA and mTA were associated with greater disability at 12 months. Structural MRI may therefore provide complementary information for prognostic assessment, pending prospective validation.

## Introduction

Autoimmune encephalitis (AE) is a severe immune-mediated inflammatory disorder of the central nervous system. While the discovery of autoantibodies targeting neuronal surface proteins has transformed the diagnosis of AE (1–4), a substantial proportion of patients presenting with typical limbic or diffuse encephalopathy remain antibody-negative despite comprehensive serological testing, thereby posing a persistent prognostic and therapeutic challenge (5–8).

The clinical spectrum of autoimmune encephalitis (AE) is remarkably diverse, ranging from classical limbic encephalitis to diffuse encephalopathy with prominent neurologic and psychiatric manifestations (9, 10). While specific antibody-positive syndromes—such as anti-NMDAR and anti-LGI1 encephalitis—have been well characterized (11, 12), the clinical features of antibody-negative cases remain poorly understood (13, 14). Crucially, comparative data regarding the natural history, relapse trajectories, and long-term functional outcomes between antibody-positive and antibody-negative AE cohorts remain scarce.

Disease relapse remains a primary driver of cumulative neurological disability in AE. Although previous studies indicate varying relapse rates among distinct antibody-mediated syndromes (15, 16), predictive risk stratification currently relies heavily on heterogeneous clinical cohorts. Consequently, it remains unclear whether the specific clinical predictors of recurrence diverge fundamentally between antibody-positive and antibody-negative patients, or across distinct antibody subtypes. Concurrently, despite immunotherapy serving as the cornerstone of AE management, its efficacy in preventing relapses—particularly in the antibody-negative population—remains contentious (5, 8, 17–19).Beyond clinical recurrence, although acute neuroimaging abnormalities are widely recognized, the longitudinal impact of macroscopic structural brain injury—specifically regional atrophy—on functional recovery remains is largely unexplored. It remains unknown whether such structural degradation differs between antibody-positive and antibody-negative cohorts, and how it intersects with disease relapse to dictate permanent disability.

We hypothesized that while antibody-positive and antibody-negative AE exhibit divergent baseline clinical profiles and subtype-specific relapse predictors, downstream structural brain atrophy serves as a convergent, antibody-independent determinant of long-term functional decline. To test this hypothesis, we conducted a large-scale, multicenter cohort study to systematically define the clinical phenotypes, distinct relapse predictors, and neuroimaging structural correlates that drive clinical outcomes across the serological spectrum of AE.

## Methods

### Study design and participants

We conducted a multicenter, retrospective cohort study of patients diagnosed with autoimmune encephalitis across eleven tertiary medical centers between January 2016 and October 2024. Diagnoses were established strictly according to the 2016 clinical diagnostic criteria for AE and independently verified by two neurologists (20). Antibody-positive AE was defined by the detection of neuronal surface antibodies (NMDAR, LGI1, GABABR, CASPR2, mGluR5, DPPX, AMPAR1, IgLON5, and GlyR) in cerebrospinal fluid (CSF) or serum using standardized cell- and tissue-based assays. Antibody-negative AE was defined by the absence of both neuronal surface and intracellular autoantibodies on comprehensive serological and CSF screening, meeting the criteria for probable or definite antibody-negative AE (20). Serum and CSF samples were collected at disease onset prior to immunotherapy initiation.

Exclusion criteria comprised (1): alternative etiologies including infectious, toxic, metabolic, or genetic disorders, specifically Creutzfeldt-Jakob disease, viral encephalitis, and febrile infection-related epilepsy syndrome (2); concurrent central nervous system demyelinating disorders defined by seropositivity for AQP4, MOG, or GFAP antibodies (3); intracellular or onconeural antibody positivity (including anti-Hu, anti-Yo, anti-Ri, anti-CV2, anti-amphiphysin, anti-CRMP5, or anti-Ma2) (4); systemic autoimmune diseases (including systemic lupus erythematosus, antiphospholipid syndrome, vasculitis, or sarcoidosis) (5); preexisting severe neurological disabilities confounding outcome assessments (6); incomplete baseline clinical or neuroimaging data; or (7) follow-up duration of less than 12 months. The study protocol was approved by the Ethics Committee of the Affiliated Hospital of Xuzhou Medical University (XYFY2024-KL623-02), and written informed consent was obtained from all participants or their legal representatives. The study also complies with the Declaration of Helsinki.

### Clinical and neurophysiological assessments

Baseline data encompassing demographics, clinical phenotypes, neurological findings, and ancillary investigations were systematically extracted. Disease severity and functional disability were quantified at admission and longitudinally using the Clinical Assessment Scale in Autoimmune Encephalitis (CASE) and the modified Rankin Scale (mRS). Standardized 21-channel continuous video-EEG (≥12 hours; international 10–20 system) was performed. Two blinded, board-certified electroencephalographers independently graded findings, including epileptiform discharges and diffuse slow-wave activity (0.5–4 Hz).

### Structural MRI assessment of brain atrophy

Cranial MRI scans obtained during routine clinical care were retrospectively reviewed, including scans acquired during the acute phase, at relapse, and at scheduled 3-, 6-, and 12-month follow-up visits. Atrophy was assessed on all available MRI scans. Because scan timing varied across patients, associations between atrophy and 12-month functional outcome were interpreted as cross-sectional and noncausal. Diffuse cortical atrophy (DCA) and medial temporal atrophy (mTA) were evaluated on axial T1-weighted images, and cerebellar hemispheric atrophy (CHA) on sagittal T1-weighted sequences. Atrophy severity was graded using established 4-point visual rating scales, with grade 1 classified as mild and grades 2–3 as moderate-to-severe atrophy (21–23). Two independent raters (a neurologist and a neuroradiologist) blinded to clinical outcomes and antibody serostatus scored all scans independently, and discrepancies were resolved by consensus.

### Definitions and follow-up procedures

Encephalitis relapse was defined as the new onset or significant worsening of neurological symptoms occurring after a clinically stable remission period of ≥1 month (20, 24). Delayed treatment was defined as immunotherapy initiation >30 days post-symptom onset (25). First-line immunotherapy included corticosteroids, intravenous immunoglobulin (IVIG), and/or plasma exchange. Second-line regimens included rituximab or intravenous cyclophosphamide. Long-term maintenance therapy was defined as azathioprine or mycophenolate mofetil usage for ≥12 months. Follow-up evaluations for encephalitis relapse were systematically conducted using inpatient and outpatient electronic medical records or via telephone interviews. All clinical assessments were performed by the treating physicians, who had completed standardized, study-specific training prior to the evaluations. The follow-up period was terminated upon patient death or loss to follow-up.

### Statistical analysis

All statistical analyses were performed using SPSS version 27.0 (IBM Corp., Armonk, NY, USA) and R software (version 4.5.2). Continuous variables, which were non-normally distributed, were expressed as medians [P25, P75] and compared using the Mann-Whitney *U* test for independent cross-sectional group comparisons. Paired ordinal data (CASE and mRS scores at baseline and relapse) were compared using the Wilcoxon signed-rank test. Longitudinal changes in clinical severity (CASE and mRS scores) across multiple follow-up time points were evaluated using the Friedman test, followed by *post-hoc* pairwise comparisons with Bonferroni correction. Categorical variables were presented as frequencies (%) and analyzed using the Chi-square or Fisher’s exact test. Missing data, restricted to 4% for CSF parameters, were imputed using the median value of the corresponding variable.

To identify independent predictors of relapse, candidate variables—including delayed immunotherapy, tumor presence, CSF protein level, sex, clinical phenotype, slow-wave activity, interictal discharges, seizure frequency, and FBDS—demonstrating *P* < 0.20 in univariable analyses were incorporated into multivariable logistic regression models. Model stability was internally validated utilizing 1,000-iteration bootstrap resampling, and results were reported as odds ratios (ORs) with 95% confidence intervals (CIs). To account for death as a competing event, Fine–Gray regression was additionally performed. Given that FBDS occurred exclusively in specific subtypes, a restricted multivariable regression was *a priori* planned for the LGI1-seropositive subgroup to adjust for subtype-specific confounders.

Comparisons of treatment response and structural neuroimaging outcomes between antibody-positive and antibody-negative cohorts were performed using the aforementioned nonparametric tests for cross-sectional and longitudinal measures. Inter-rater reliability for DCA, mTA, and CHA, each rated on an ordinal 4-point scale (0–3), was assessed separately by antibody serostatus. Linearly weighted Cohen κ was used as the primary reliability measure to account for the ordinal scale structure, and quadratically weighted κ was calculated in sensitivity analyses. Ninety-five percent CIs were estimated using the asymptotic normal approximation. Exact agreement and disagreements limited to adjacent categories were additionally reported to facilitate interpretation, particularly for skewed score distributions. All tests were 2-sided, with *P* < 0.05 considered statistically significant.

## Results

### Patient selection and antibody distribution

**Figure 1:**
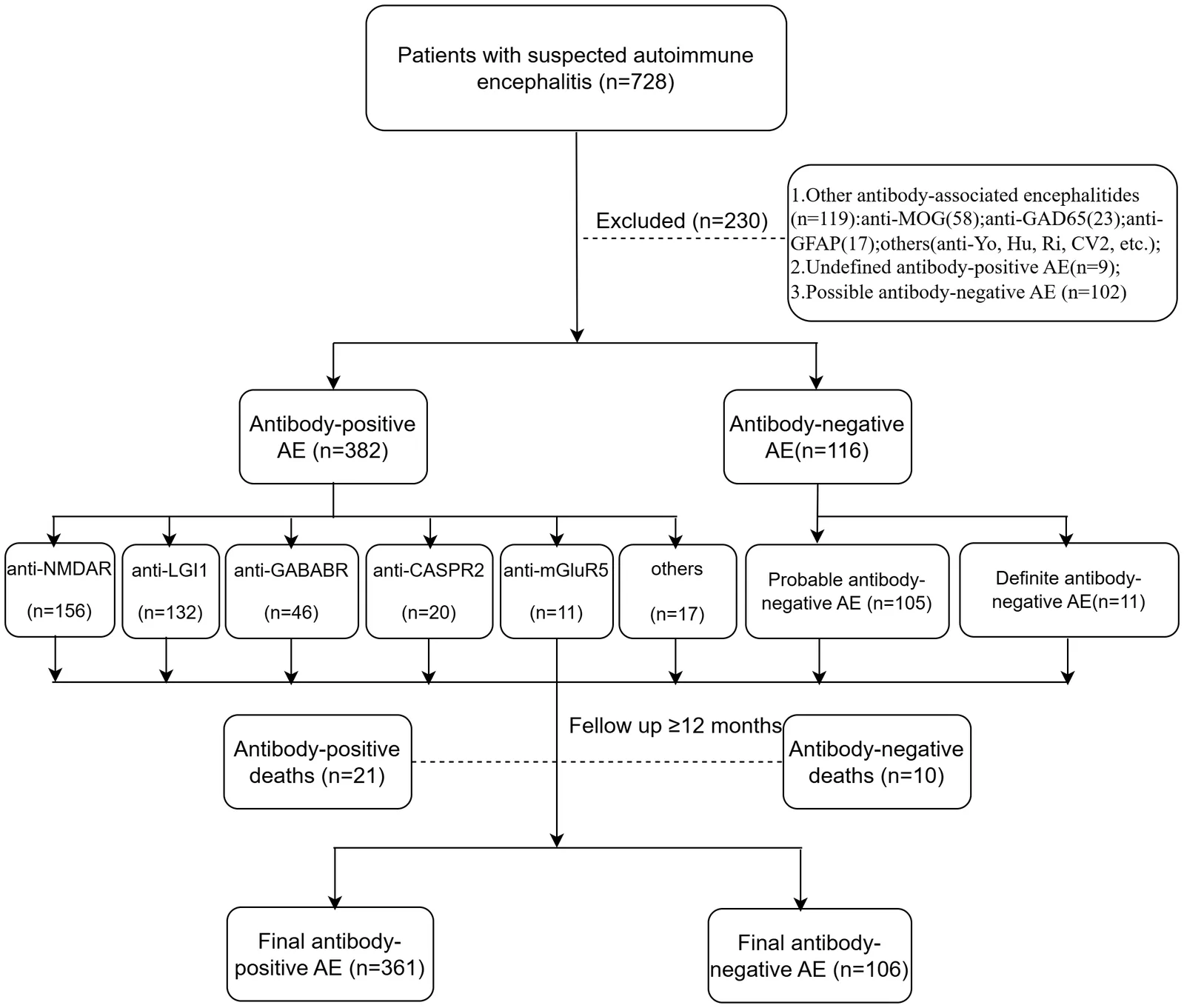
Flowchart illustrating the design and patient selection of the autoimmune encephalitis (AE) cohort.

Among 728 patients evaluated for suspected AE, 230 were excluded (**Figure 1**). Exclusions comprised 119 patients with non-neuronal surface antibody–associated encephalitis [anti-MOG (n=58), anti-GAD65 (n=23), anti-GFAP (n=17), anti-Yo (n=3), anti-Hu (n=2), anti-Drebrin (n=2), anti-Ri (n=2), anti-CV2 (n=2), and single cases each of anti-recoverin, anti-Ma2, anti-ITPR1, anti-AK5, anti-GM2, anti-Homer3, anti-ARHGAP26, anti-amphiphysin, anti-NF-H, and anti-AGO], 9 with undefined antibody profiles (antibody negative on cell-based assays but positive on tissue-based immunofluorescence), and 102 with possible AE who did not meet the diagnostic criteria for probable or definite AE.

The final study cohort consisted of 498 patients. This included 382 (76.7%) patients with neuronal surface antibody–positive AE and 116 (23.3%) patients with antibody-negative AE (comprising 105 probable and 11 definite cases). Within the antibody-positive subgroup (n=382), the most frequent specificities were anti-NMDAR (n=156, 40.8%) and anti-LGI1 (n=132, 34.6%), followed by anti-GABABR (n=46, 12.0%), anti-CASPR2 (n=20, 5.2%), and anti-mGluR5 (n=11, 2.9%); the remaining 17 cases (4.5%) involved rare specificities. During follow-up, 31 patients (6.2%) died, including 21 in the antibody-positive group and 10 in the antibody-negative group. The remaining 467 patients (93.8%) completed the designated follow-up.

### Clinical, radiologic, and immunologic characteristics

The cohort comprised 498 patients with AE, including 382 antibody-positive and 116 antibody-negative cases (**Table 1**). At baseline, the median age was 52 years [28, 65] in the antibody-positive group and 50 years [26, 62] in the antibody-negative group, with females accounting for 43.5% (n = 166) and 36.2% (n = 42), respectively. Daily or multiple seizures were observed in 35.6% of antibody-positive and 25.9% of antibody-negative patients (*P* = 0.051). Faciobrachial dystonic seizures (FBDS) occurred exclusively in the antibody-positive group (*P* < 0.001). On MRI, antibody-negative patients exhibited higher frequencies of diffusion restriction across cortical (53.4% vs 20.4%), subcortical (41.4% vs 12.0%), medial temporal (41.4% vs 30.4%), and infratentorial regions (9.5% vs 3.9%) (all *P* < 0.05). Cerebrospinal fluid analysis revealed higher leukocyte counts (median 10 vs 3 ×10^6^/L, *P* < 0.001) and lower protein levels (median 0.38 vs 0.45 g/L, *P* = 0.008) in antibody-positive patients. Regarding treatment, antibody-positive patients more frequently received combined first-line therapy, maintenance regimens, and second-line immunotherapy (all *P* < 0.001). Baseline CASE score was statistically higher in the antibody-positive cohort (median 5 [4, 7] vs 5 [3, 6], *P* = 0.049), whereas mortality rates remained comparable (5.5%vs8.6%, *P* = 0.223). During follow-up, 31 of 498 patients (6.2%) died. Compared with survivors (n=467), deceased patients were older at onset (67.0 [49.0, 72.0] vs 51.0 [27.0, 63.0], *P* < 0.001, **Supplementary Table 1**), more frequently had a baseline GCS score ≤13 (51.6% vs 28.7%, *P* = 0.007), and were more often admitted to the ICU (32.3% vs 15.8%, *P* = 0.018). Tumors were also more prevalent among deceased patients (32.3% vs 7.1%, *P* < 0.001), who had higher baseline mRS scores (4 [3, 4] vs 3 [2, 3], *P* = 0.002). Conversely, focal seizures were less frequent in patients who died (54.8% vs 72.6%, *P* = 0.034). Excluded possible AE cases (n=102) were comparable with probable antibody-negative AE cases (n=116) in demographic characteristics, initial presentation, clinical severity, and immunotherapy (**Supplementary Table 2**). Possible AE cases had less frequent MRI abnormalities and lower CSF leukocyte counts (3.0 vs 10.0×10^6^/L, *P* < 0.001), consistent with insufficient objective inflammatory evidence to fulfill criteria for probable AE, rather than systematic differences in clinical phenotype or treatment.

**Table 1:** Clinical characteristics of the patients.

Characteristics	Antibody positive (n=382)	Antibody negative (n=116)	*P*
Female (%)	166 (43.5)	42 (36.2)	0.166
Age at onset (range), y	52 (3–87)	49.5 (4-80)	0.233
Initial presentation			
Neuropsychiatric	95 (24.7)	34 (29.3)	0.339
Seizures	167 (43.7)	40 (34.5)	0.077
Memory dysfunction	78 (20.4)	27 (23.3)	0.509
Impaired consciousness	11 (2.9)	5 (4.3)	0.642
Language problem	12 (3.1)	4 (3.4)	1.000
Dyskinesia/dystonia	8 (2.1)	2 (1.7)	1.000
Gait instability and ataxia	3 (0.8)	3 (2.6)	0.142
Other	8 (2.1)	1 (0.9)	0.635
Delayed immunotherapy (%)	124 (32.5)	36 (31.0)	0.773
≥2ASM	138 (36.1)	40 (34.5)	0.746
ICU admission (%)	60 (15.7)	24 (20.7)	0.209
GCS scores ≤ 13 (%)	108 (28.3)	42 (36.2)	0.103
Any cognitive concerns, n (%)	334 (87.4)	100 (86.2)	0.729
Presence of tumor (%)	35 (9.2)	8 (6.9)	0.447
Diffusion restriction (%)			
Cortex (%)	78 (20.4)	62 (53.4)	<0.001
Subcortex/white matter (%)	46 (12.0)	48 (41.4)	<0.001
Medial temporal cortex (%)	116 (30.4)	48 (41.4)	0.027
Infra-tentorium (%)	15 (3.9)	11 (9.5)	0.018
Video-EEG			
Diffuse slow wave	87 (22.8)	31 (26.7)	0.381
Interictal epileptiform discharge	195 (51.0)	56 (48.3)	0.601
Seizures captured	53 (13.9)	15 (12.9)	0.796
Initially treated with combined first-line IT, n (%)	242 (63.3)	51 (43.9)	<0.001
Initially treated with long-term IT, n (%)	70 (18.3)	4 (3.4)	<0.001
Initially treated with second-line IT, n (%)	31 (8.1)	0 (0.0)	<0.001
Treated with long-term IT during course, n (%)	74 (19.4)	5 (4.3)	<0.001
Treated with second-line IT during course, n (%)	22 (5.8)	2 (1.7)	0.076
Highest seizure frequency, n (%)			
Single seizure Multiple daily/daily Weekly Monthly	22 (5.8)	5 (4.3)	0.546
	136 (35.6)	30 (25.9)	0.051
	91 (23.8)	28 (24.1)	0.944
	40 (10.5)	12 (10.3)	0.969
Type of seizure at onset, n (%)			
FBDS Focal seizures SE	39 (10.2)	0 (0.0)	<0.001
	281 (73.6)	75 (64.7)	0.063
	61 (16.0)	26 (22.4)	0.109
CSF leucocyte level (10^6^/L) M (P25,P75)	10.0 (2.0,22.0)	3.0 (2.0,9.8)	<0.001
CSF protein level (g/L) M (P25,P75)	0.38 (0.28,0.56)	0.45 (0.30, 0.66)	0.008
Baseline CASE scores M (P25,P75)	5 (4,7)	5 (3,6)	0.049
Baseline mRS scores M (P25,P75)	3 (2,3.25)	3 (2,4)	0.455
Mortality rate, n (%)	21 (5.5)	10 (8.6)	0.223

ASM, antiseizure medication; ICU, intensive care unit; EEG, electroencephalography; IT, immunotherapy; FBDS, faciobrachial dystonic seizures; SE, status epilepticus; CASE, Clinical Assessment Scale in Autoimmune Encephalitis; mRS, modified Rankin Scale.

### Predictors of relapse in autoimmune encephalitis

**Figure 2:**
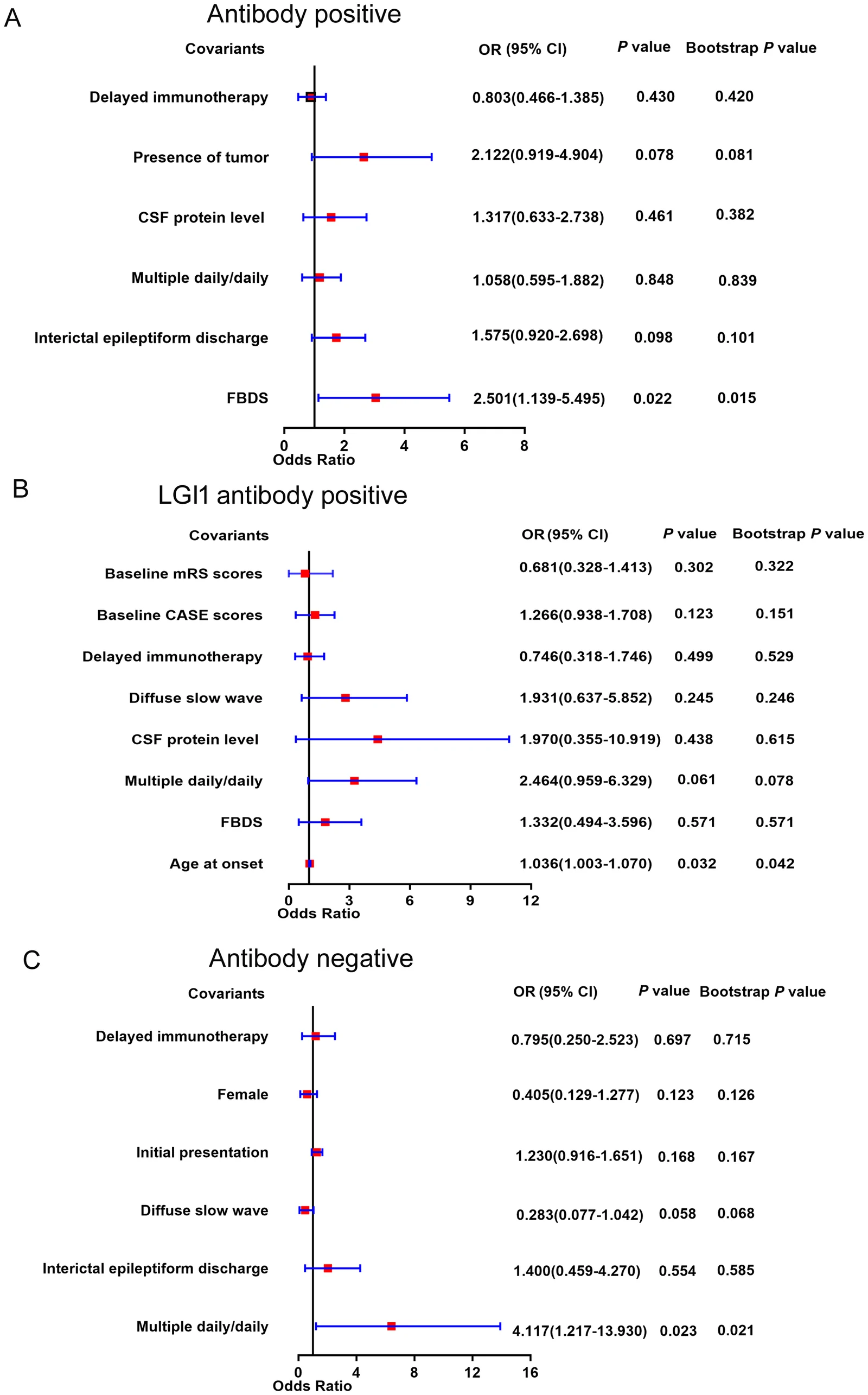
Independent predictors of relapse in autoimmune encephalitis. **(A)** Forest plot of multivariable logistic regression analysis for predictors of relapse in antibody-positive autoimmune encephalitis. **(B)** Forest plot of multivariable logistic regression analysis for predictors of relapse in LGI1 antibody-positive autoimmune encephalitis. **(C)** Forest plot of multivariable logistic regression analysis for predictors of relapse in antibody-negative autoimmune encephalitis. Data are presented as odds ratios (ORs) with 95% confidence intervals (CIs). Model stability was internally validated using 1,000 bootstrap resamples. OR, odds ratio; CI, confidence interval; FBDS, faciobrachial dystonic seizures; mRS, modified Rankin Scale; CASE, Clinical Assessment Scale in Autoimmune Encephalitis.

Among antibody-positive patients, 92 (25.5%) experienced at least one relapse, including those with anti-LGI1 (n = 40), anti-NMDAR (n = 40), anti-CASPR2 (n = 5), anti-GABABR (n = 4), and single rare subtypes. Multiple recurrences were observed in 18 patients (5.0%). In univariable analysis, relapse was associated with interictal epileptiform discharges (60.9% vs 47.6%, *P* = 0.028), FBDS (17.4% vs 7.8%, *P* = 0.009), and elevated CSF protein levels (0.47 vs 0.35 g/L, *P* = 0.013) (**Table 2**). In multivariable logistic regression with 1,000 bootstrap replications, FBDS was independently associated with relapse (OR = 2.50, 95% CI 1.14–5.50, *P* = 0.022, **Figure 2A**). To account for death as a competing event, Fine–Gray regression was additionally performed in the 382-patient antibody-positive AE cohort, in which 92 patients relapsed and 21 died during follow-up. In the multivariable model, FBDS was independently associated with a higher cumulative incidence of relapse (subdistribution hazard ratio [sHR]=1.91, 95% CI 1.04–3.52, *P* = 0.038, **Supplementary Figure 1**), whereas higher CSF leukocyte levels were associated with a lower relapse incidence (sHR=0.99, 95% CI 0.98–1.00, *P* = 0.030).

**Table 2:** Comparative characteristics of patients with antibody-positive encephalitis: relapsing versus non-relapsing cases.

Characteristics	Relapse (n=92)	No relapse (n=269)	*P*
Female (%)	41 (44.6)	117 (43.5)	0.858
Age at onset (range), y	52.5 (6-83)	52 (3-87)	0.717
Initial presentation			
Neuropsychiatric	24 (26.1)	66 (24.5)	0.766
Seizures	42 (45.7)	115 (42.8)	0.628
Memory dysfunction	18 (19.6)	56 (20.8)	0.797
Impaired consciousness	1 (1.1)	10 (3.7)	0.360
Language problem	2 (2.2)	10 (3.7)	0.707
Dyskinesia/dystonia	1 (1.1)	5 (1.9)	1.000
Gait instability and ataxia	0 (0.0)	3 (1.1)	0.574
Other	4 (4.3)	4 (1.5)	0.231
Delayed immunotherapy (%)	28 (30.4)	86 (32.0)	0.784
≥2ASM	33 (35.9)	98 (36.4)	0.923
ICU admission (%)	12 (13.0)	40 (14.9)	0.667
GCS scores ≤ 13 (%)	21 (22.8)	77 (28.6)	0.280
Any cognitive concerns, n (%)	81 (88.0)	236 (87.7)	0.937
Presence of tumor (%)	10 (10.9)	17 (6.3)	0.152
Diffusion restriction (%)			
Cortex (%)	23 (25.0)	53 (19.7)	0.282
Subcortex/white matter (%)	12 (13.0)	33 (12.3)	0.846
Medial temporal cortex (%)	32 (34.8)	80 (29.7)	0.367
Infra-tentorium (%)	3 (3.3)	12 (4.5)	0.845
Video-EEG			
Diffuse slow wave	22 (23.9)	61 (22.7)	0.808
Interictal epileptiform discharge	56 (60.9)	128 (47.6)	0.028
Seizures captured	16 (17.4)	35 (13.0)	0.298
Initially treated with combined first-line IT, n (%)	55 (59.8)	174 (64.7)	0.399
Initially treated with long-term IT, n (%)	18 (19.6)	48 (17.8)	0.712
Initially treated with second-line IT, n (%)	9 (9.8)	20 (7.4)	0.475
Treated with long-term IT during course, n (%)	20 (21.7)	51 (19.0)	0.563
Treated with second-line IT during course, n (%)	6 (6.5)	15 (5.6)	0.738
combined first-line IT+long-term IT during course	14 (15.2)	36 (13.4)	0.660
combined first-line IT + second-line IT during course	2 (2.2)	15 (5.6)	0.296
Highest seizure frequency, n (%)			
Single seizure Multiple daily/daily Weekly Monthly	2 (2.2)	19 (7.1)	0.084
	40 (43.5)	89 (33.1)	0.073
	22 (23.9)	67 (24.9)	0.849
	10 (10.9)	25 (9.3)	0.659
Type of seizure at onset, n (%)			
FBDS Focal seizures SE	16 (17.4)	21 (7.8)	0.009
	73 (79.3)	197 (73.2)	0.244
	17 (18.5)	39 (14.5)	0.363
CSF leucocyte level (10^6^/L) M (P25,P75)	9.0 (3.0,15.0)	10.0 (2.0,27.0)	0.274
CSF protein level (g/L) M (P25,P75)	0.47 (0.30,0.56)	0.35 (0.27,0.54)	0.013
Baseline CASE scores M (P25,P75)	6 (4,7)	5 (3,7)	0.277
Immunotherapy-3M CASE scores M (P25,P75)	2 (2,3)	2 (1,3)	0.504
Baseline mRS scores M (P25,P75)	3 (2,3)	3 (2,4)	0.723
Immunotherapy-3M mRS scores M (P25,P75)	2 (1,2)	2 (1,2)	0.851

ASM, antiseizure medication; ICU, Intensive Care Unit; IT, immunotherapy; FBDS, faciobrachial dystonic seizures; SE, status epilepticus; CASE, Clinical Assessment Scale in Autoimmune Encephalitis; mRS, modified Rankin Scale.

Relapse rates varied by antibody subtype: 34.8% in the anti-LGI1 group, 26.8% in the anti-NMDAR group, 26.3% in the anti-CASPR2 group, and 9.8% in the anti-GABABR group. FBDS was observed exclusively in patients with LGI1-antibody encephalitis. Within this LGI1 subgroup, the frequency of FBDS was 40.0% in relapsing patients and 24.7% in non-relapsing patients (*P* = 0.086, **Table 3**). In multivariable regression restricted to the LGI1 cohort, older age at onset was independently associated with relapse (OR = 1.04, 95% CI 1.00–1.07, *P* = 0.042, **Figure 2B**), whereas FBDS was not. Patients aged >65 years received combined first-line immunotherapy less frequently than younger patients (44.7% vs 66.9%, P = 0.010) and showed a numerically higher prevalence of concomitant tumors (8.5% vs 1.3%, P = 0.066). Among older patients, treatment adherence, concomitant tumors, and maintenance immunotherapy during follow-up did not differ between those with and without relapse (all *P*>0.05, **Supplementary Table 3**), and none was independently associated with relapse in multivariable models. Consistently, no interaction was observed between age group and these variables for relapse risk (all *P*_interaction_>0.05).

**Table 3:** Comparative characteristics of patients with LGI 1 antibody-positive encephalitis: relapsing versus non-relapsing cases.

Characteristics	Relapse (n=40)	No relapse (n=85)	*P*
Female (%)	18 (45.0)	38 (44.7)	0.975
Age at onset (range), y	64.5 (17-81)	59 (18-87)	0.012
Initial presentation			
Neuropsychiatric	7 (17.5)	8 (9.4)	0.240
Seizures	23 (57.5)	48 (56.5)	0.914
Memory dysfunction	8 (20.0)	28 (32.9)	0.136
Other	2 (5.0)	1 (1.2)	0.240
Delayed immunotherapy (%)	19 (47.5)	41 (48.2)	0.939
≥2ASM	12 (30.0)	32 (37.6)	0.404
ICU admission (%)	5 (12.5)	3 (3.5)	0.109
GCS scores ≤ 13 (%)	8 (20.0)	15 (17.6)	0.894
Any cognitive concerns, n (%)	34 (85.0)	73 (85.9)	0.896
Presence of tumor (%)	3 (7.5)	2 (2.4)	0.326
Diffusion restriction (%)			
Cortex (%)	3 (7.5)	12 (14.1)	0.383
Subcortex/white matter (%)	0 (0.0)	4 (4.7)	0.305
Medial temporal cortex (%)	14 (35.0)	34 (40.0)	0.592
Infra-tentorium (%)	0 (0.0)	3 (3.5)	0.551
Video-EEG			
Diffuse slow wave	10 (25.0)	10 (11.8)	0.060
Interictal epileptiform discharge	25 (62.5)	45 (52.9)	0.315
Seizures captured	5 (12.5)	12 (14.1)	0.806
Initially treated with combined first-line IT, n (%)	22 (55.0)	52 (61.2)	0.512
Initially treated with long-term IT, n (%)	9 (22.5)	13 (15.3)	0.324
Initially treated with second-line IT, n (%)	2 (5.0)	1 (1.2)	0.240
Treated with long-term IT during course, n (%)	10 (25.0)	14 (16.5)	0.259
Treated with second-line IT during course, n (%)	2 (5.0)	3 (3.5)	0.655
combined first-line IT+long-term IT during course	7 (17.5)	8 (9.4)	0.240
combined first-line IT + second-line IT during course	2 (5.0)	3 (3.5)	0.655
Highest seizure frequency, n (%)			
Single seizure Multiple daily/daily Weekly Monthly	1 (2.5)	4 (4.7)	1.000
	24 (60.0)	34 (40.0)	0.036
	8 (20.0)	26 (30.6)	0.215
	2 (5.0)	9 (10.6)	0.500
Type of seizure at onset, n (%)			
FBDS Focal seizures SE	16 (40.0)	21 (24.7)	0.081
	35 (87.5)	71 (83.5)	0.564
	7 (17.5)	8 (9.4)	0.240
CSF leucocyte level (10^6^/L) M (P25,P75)	3.0 (2.0,8.0)	3.0 (2.0,6.8)	0.721
CSF protein level (g/L) M (P25,P75)	0.45 (0.30,0.58)	0.32 (0.26,0.51)	0.018
Baseline CASE scores M (P25,P75)	5 (4,6)	4 (3,6)	0.018
Immunotherapy-3M CASE scores M (P25,P75)	2 (1.25,2)	2 (1,2)	0.146
Baseline mRS scores M (P25,P75)	3 (2,3)	2 (2,3)	0.092
Immunotherapy-3M mRS scores M (P25,P75)	1 (1,2)	1 (1,2)	0.925

ASM, antiseizure medication; ICU, Intensive Care Unit; IT, immunotherapy; FBDS, faciobrachial dystonic seizures; SE, status epilepticus; CASE, Clinical Assessment Scale in Autoimmune Encephalitis; mRS, modified Rankin Scale.

Among patients with antibody-negative AE, 24 of 106 (22.6%) experienced relapse, including 2 with multiple recurrences (**Table 4**). Frequent daily seizures were independently associated with relapse in the multivariable logistic regression model (OR = 4.12, 95% CI 1.22–13.93, *P* = 0.023, **Figure 2C**). To account for death as a competing event, Fine–Gray regression was additionally performed in the 116-patient antibody-negative AE cohort, in which 24 patients relapsed and 10 died during follow-up. Multiple daily seizures remained independently associated with a higher cumulative incidence of relapse (sHR=3.05, 95% CI 1.26–7.40, *P* = 0.014, **Supplementary Figure 2**).

**Table 4:** Comparative characteristics of patients with antibody-negative encephalitis: relapsing versus non-relapsing cases.

Characteristics	Relapse (n=24)	No relapse (n=82)	*P*
Female (%)	5 (20.8)	32 (39.0)	0.100
Age at onset (range), y	53 (4-78)	43.5 (9-80)	0.472
Initial presentation			
Neuropsychiatric	7 (29.2)	24 (29.3)	0.992
Seizures	10 (41.7)	28 (34.1)	0.499
Memory dysfunction	3 (12.5)	23 (28.0)	0.119
Impaired consciousness	0 (0.0)	4 (4.9)	0.572
Language problem	0 (0.0)	2 (2.4)	1.000
Dyskinesia/dystonia	1 (4.2)	0 (0.0)	0.226
Gait instability and ataxia	2 (8.3)	1 (1.2)	0.128
Other	1 (4.2)	0 (0.0)	0.226
Delayed immunotherapy (%)	6 (0.25)	28 (34.1)	0.398
≥2ASM	10 (41.7)	25 (30.5)	0.306
ICU admission (%)	7 (29.2)	15 (18.3)	0.385
GCS scores ≤ 13 (%)	8 (33.3)	28 (34.1)	0.941
Any cognitive concerns, n (%)	20 (83.3)	71 (86.6)	0.945
Presence of tumor (%)	1 (4.2)	5 (6.1)	1.000
Diffusion restriction (%)			
Cortex (%)	14 (58.3)	42 (51.2)	0.539
Subcortex/white matter (%)	8 (33.3)	36 (43.9)	0.355
Medial temporal cortex (%)	7 (29.2)	35 (42.7)	0.234
Infra-tentorium (%)	3 (12.5)	5 (6.1)	0.545
Video-EEG			
Diffuse slow wave	4 (16.7)	26 (31.7)	0.150
Interictal epileptiform discharge	14 (58.3)	35 (42.7)	0.176
Seizures captured	4 (16.7)	10 (12.2)	0.821
Initially treated with combined first-line IT, n (%)	12 (50.0)	34 (41.5)	0.458
Initially treated with long-term IT, n (%)	0 (0.0)	4 (4.9)	0.572
Treated with long-term IT during course, n (%)	2 (8.3)	3 (3.7)	0.317
Treated with second-line IT during course, n (%)	0 (0.0)	2 (2.4)	1.000
combined first-line IT+long-term IT during course	2 (8.3)	2 (2.4)	0.220
combined first-line IT + second-line IT during course	0 (0.0)	1 (1.2)	1.000
Highest seizure frequency, n (%)			
Single seizure Multiple daily/daily Weekly Monthly	0 (0.0)	5 (6.1)	0.586
	11 (45.8)	17 (20.7)	0.014
	4 (16.7)	20 (24.4)	0.427
	2 (8.3)	10 (12.2)	0.874
Type of seizure at onset, n (%)			
Focal seizures	17 (70.8)	52 (63.4)	0.502
SE	7 (29.2)	16 (19.5)	0.313
CSF leucocyte level (10^6^/L) M (P25,P75)	3.5 (2.0,10.0)	3.0 (1.0,7.0)	0.439
CSF protein level (g/L) M (P25,P75)	0.47 (0.29,0.66)	0.42 (0.30,0.65)	0.899
CASE scores M (P25,P75)	5.0 (3.0,5.8)	5.0 (3.0,6.0)	0.403
Immunotherapy-3M CASE scores M (P25,P75)	2 (1,2)	2 (1,3)	0.402
Baseline mRS scores M (P25,P75)	2.5 (2,4)	3 (2,4)	0.853
Immunotherapy-3M mRS scores M (P25,P75)	2 (1,2)	2 (1,2)	0.678

ASM, antiseizure medication; ICU, intensive care unit; EEG, electroencephalography; IT, immunotherapy; FBDS, faciobrachial dystonic seizures; SE, status epilepticus; CASE, Clinical Assessment Scale in Autoimmune Encephalitis; mRS, modified Rankin Scale.

### Immunotherapy response in antibody-positive versus antibody-negative autoimmune encephalitis

**Figure 3:**
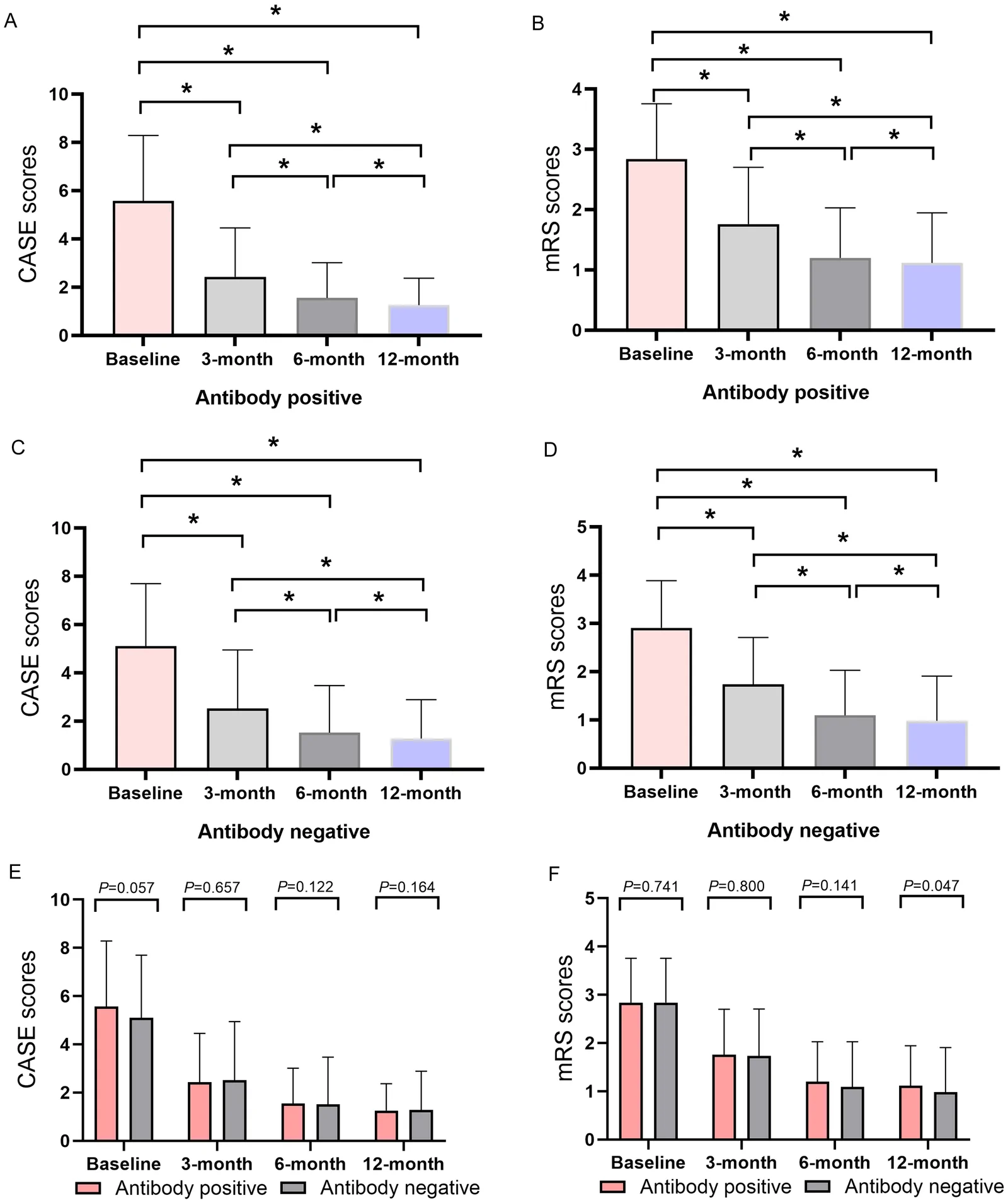
Treatment response in antibody-positive and antibody-negative patients with autoimmune encephalitis. **(A, B)** Longitudinal changes in CASE and mRS scores in antibody-positive patients at baseline and 3, 6, and 12 months after treatment. **(C, D)** Longitudinal changes in CASE and mRS scores in antibody-negative patients at baseline and 3, 6, and 12 months after treatment. **(E, F)** Between-group comparisons of CASE and mRS scores at baseline and 3, 6, and 12 months after treatment. Friedman tests were used to compare CASE and mRS scores across baseline and 3, 6, and 12 months after treatment. When the Friedman test was significant, *post hoc* pairwise comparisons were performed using Wilcoxon signed-rank tests with Bonferroni correction. Between-group comparisons of CASE and mRS scores at each time point were performed using the Mann-Whitney *U* test. CASE, Clinical Assessment Scale in Autoimmune Encephalitis; mRS, modified Rankin scale. Asterisks indicate statistical significance at a Bonferroni-adjusted *P* value < 0.05.

At baseline, the median CASE score was 5 [4, 7] in the antibody-positive group and 5 [3, 6] in the antibody-negative group (*P* = 0.057, **Figure 3E**). Baseline median mRS scores were 3 [2, 3] and 3 [2, 4], respectively (*P* = 0.741, **Figure 3F**). Following the initiation of immunotherapy, both groups exhibited progressive declines in CASE and mRS scores across all follow-up time points (all adjusted *P* < 0.05, **Figures 3A–D**). In the antibody-positive group, median CASE scores decreased to 2 [1, 3] at 3 months, 1 [1, 2] at 6 months, and 1 [1, 2] at 12 months; corresponding median mRS scores decreased to 2 [1, 2], 1 [1, 2], and 1 [1, 1]. In the antibody-negative group, median CASE scores decreased to 2 [1, 3] at 3 months, 1 [0, 2] at 6 months, and 1 [0, 1] at 12 months; corresponding median mRS scores decreased to 2 [1, 2], 1 [0, 1], and 1 [0, 1]. Cross-sectional comparisons between the two groups during follow-up revealed no statistical differences in CASE scores at 3, 6, or 12 months (all *P*>0.05, **Figure 3E**). For functional outcomes, mRS scores did not differ between the groups at 3 or 6 months (both *P*>0.05). However, at the 12-month follow-up, the antibody-negative group demonstrated statistically lower mRS scores than the antibody-positive group (median 1 [0, 1] vs 1 [1, 1], *P* = 0.047, **Figure 3F**).

### Impact of initial clinical presentation

**Figure 4:**
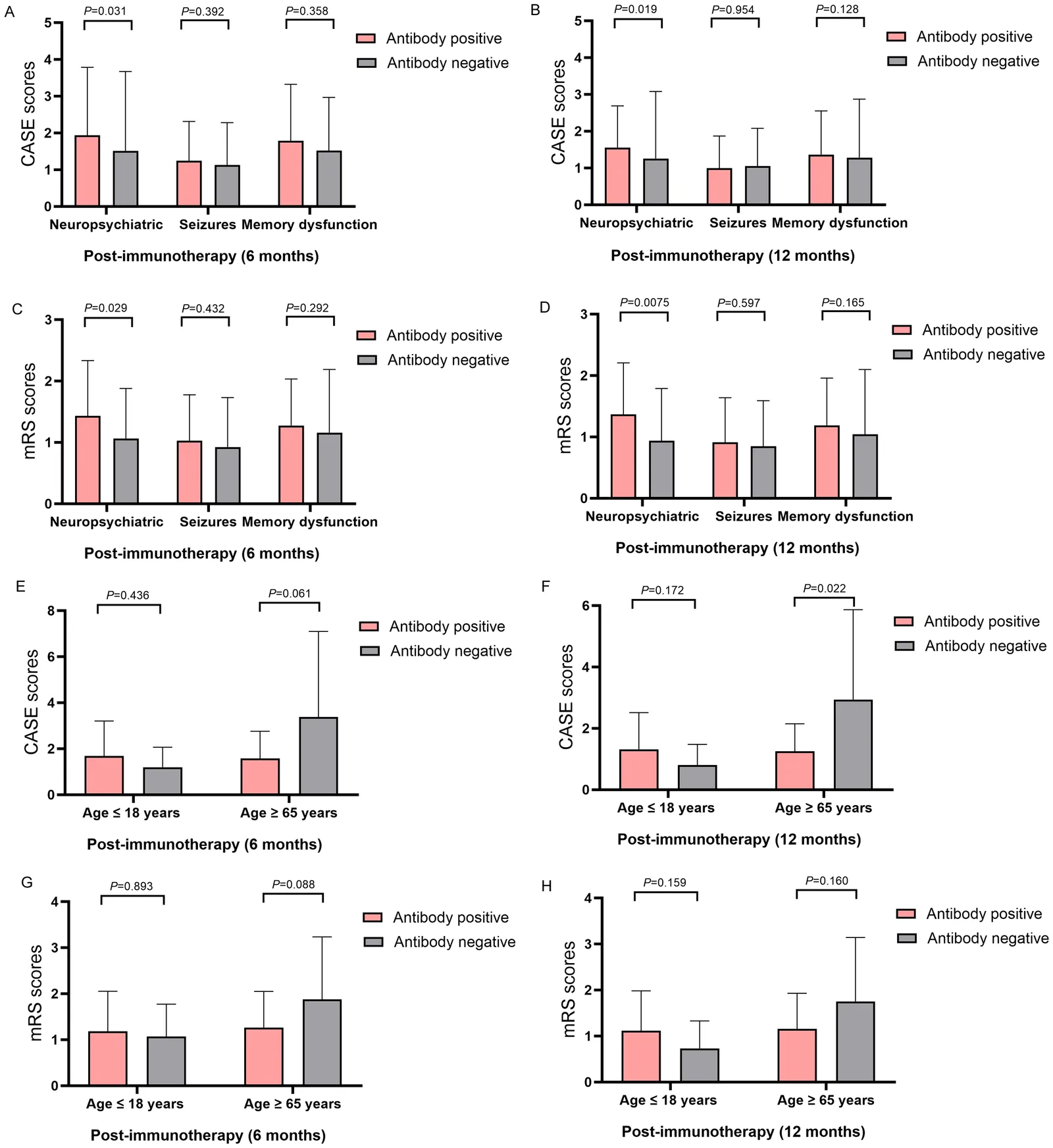
Effect of initial presentation and age on immunotherapeutic outcomes in antibody-positive and antibody-negative autoimmune encephalitis. **(A, B)** CASE scores at 6 and 12 months after immunotherapy across neuropsychiatric, seizure, and memory phenotypes in patients with antibody-positive and antibody-negative autoimmune encephalitis. **(C, D)** mRS scores at 6 and 12 months after immunotherapy across clinical phenotypes. **(E, F)** CASE scores at 6 and 12 months after immunotherapy in pediatric (≤18 years) and elderly (≥65 years) patients with antibody-positive and antibody-negative autoimmune encephalitis. **(G, H)** mRS scores at 6 and 12 months after immunotherapy in pediatric and elderly patients. Between-group comparisons were performed using the Mann-Whitney *U* test. CASE, Clinical Assessment Scale in Autoimmune Encephalitis; mRS, modified Rankin scale.

Clinical outcomes among the 467 patients who completed follow-up were evaluated based on initial symptom phenotypes. In patients with predominant psychiatric symptoms, antibody-positive cases (n = 90) exhibited higher clinical severity scores than antibody-negative cases (n = 31) at 6 months (CASE: 1 [1, 2] vs 1 [1, 2], *P* = 0.031; mRS: 1 [1, 2] vs 1 [1, 1], *P* = 0.029; **Figure 4A, C**) and at 12 months (CASE: 1 [1, 2] vs 1 [0, 1], *P* = 0.019; mRS: 1 [1, 2] vs 1 [0, 1], *P* = 0.008; **Figure 4B, D**). Conversely, in patients with seizure- or cognition-dominant presentations, CASE and mRS scores did not differ between the antibody-positive and antibody-negative groups at 6 or 12 months (all *P* > 0.05, **Figures 4A–D**).

### Age-dependent variations in outcomes

Outcomes were further evaluated in a subset of 158 patients, stratified into pediatric (≤ 18 years) and elderly (≥ 65 years) cohorts. In the pediatric cohort, CASE and mRS scores were comparable between antibody-positive (n = 44) and antibody-negative (n = 15) patients at both 6 and 12 months (all *P* > 0.05, **Figures 4E–H**).

In the elderly cohort, median baseline CASE scores were higher in antibody-negative patients (n = 16) compared with antibody-positive patients (n = 83) (7 [5, 10] vs 5 [4, 7], *P* = 0.027). Baseline mRS scores did not differ statistically between the two groups (3 [2, 4] vs 3 [2, 3], *P* = 0.180). Furthermore, there were no statistical differences between the antibody-negative and antibody-positive groups regarding the use of first-line combination therapy (25.0% vs 41.0%, *P* = 0.229), long-term immunotherapy (12.5% vs 15.7%, *P* > 0.05), delayed treatment (37.5% vs 36.1%, *P* > 0.05), or the presence of a coexisting tumor (6.3% vs 14.5%, *P* > 0.05).

At the 6-month follow-up, median CASE scores were 2 [1, 5] in the antibody-negative group and 1 [1, 2] in the antibody-positive group (*P* = 0.061, **Figure 4E**), while median mRS scores were 2 [1, 3] and 1 [1, 2], respectively (*P* = 0.088, **Figure 4G**). By 12 months, CASE scores were statistically higher in the antibody-negative group than in the antibody-positive group (2 [1, 5] vs 1 [1, 2], *P* = 0.022, **Figure 4F**). However, mRS scores at 12 months showed no statistical difference between the two cohorts (1 [1, 3] vs 1 [1, 1], *P* = 0.160, **Figure 4H**).

### Temporal patterns and severity of relapse

**Figure 5:**
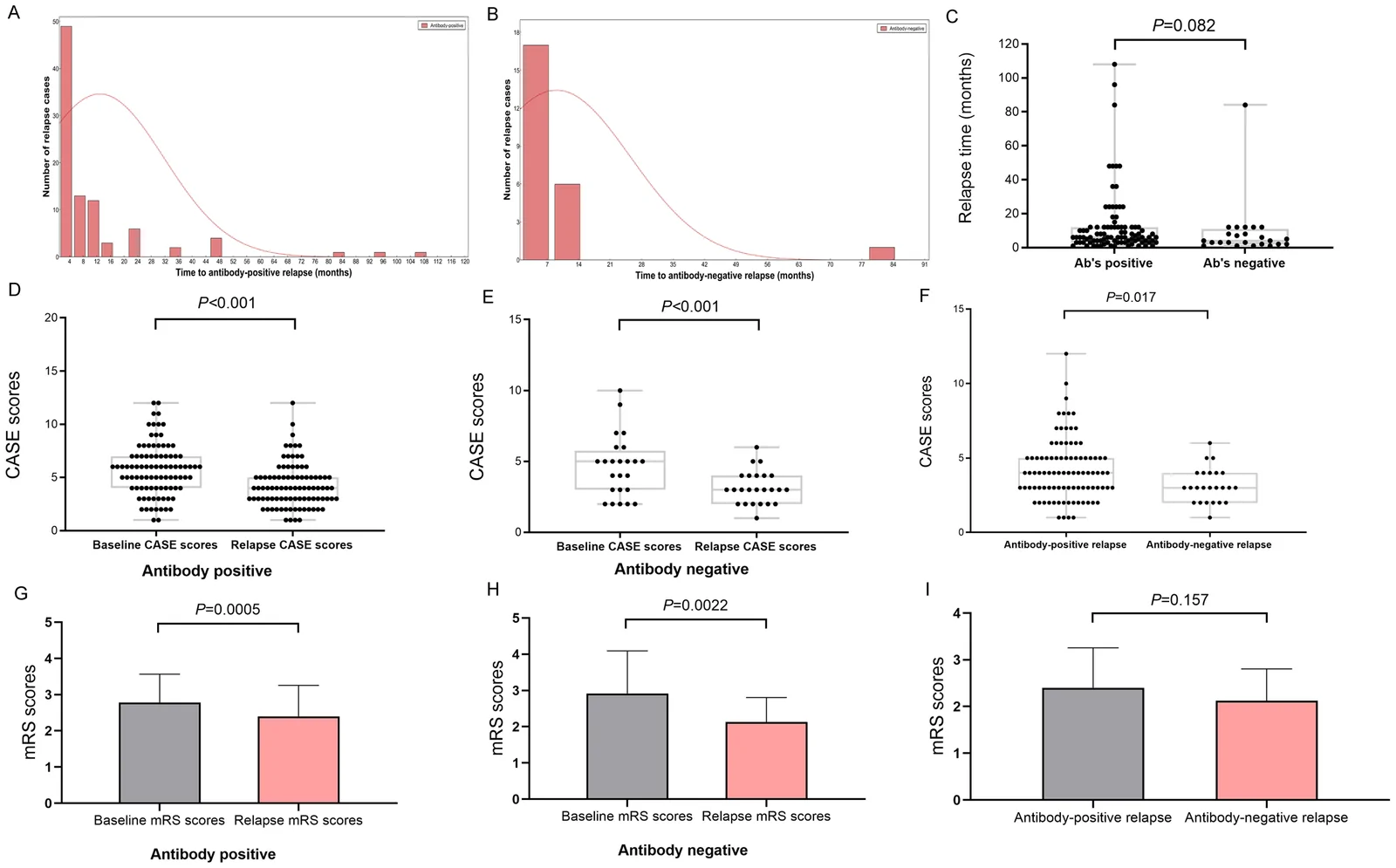
Relapse characteristics and disease severity in antibody-positive and antibody-negative autoimmune encephalitis. **(A, B)** Temporal distribution of first relapse events in the antibody-positive and antibody-negative autoimmune encephalitis cohorts. **(C)** Comparison of time to first relapse between the two cohorts. **(D, E)** Comparison of disease severity, assessed by CASE scores, between initial presentation and relapse in antibody-positive and antibody-negative patients. **(F)** Comparison of CASE scores at relapse between the two cohorts. **(G, H)** Comparison of functional impairment, assessed by mRS scores, between initial presentation and relapse in antibody-positive and antibody-negative patients. **(I)** Comparison of mRS scores at relapse between the two cohorts. Paired CASE and mRS scores at baseline and relapse were compared using the Wilcoxon signed-rank test. Between-group comparisons were performed using the Mann-Whitney *U* test. CASE, clinical assessment scale in autoimmune encephalitis; mRS, modified Rankin scale.

The median time to relapse was 6 months [3, 12] in antibody-positive patients and 4.0 months [2, 11] in antibody-negative patients (*P* = 0.082, **Figures 5A–C**). At the time of relapse, both cohorts presented with lower disease severity scores compared with their respective baselines. In the antibody-positive group, the median CASE score at relapse was 4 [3, 5], compared with 6 [4, 7] at baseline (*P* < 0.001, **Figure 5D**). The median mRS score at relapse was 2 [2, 3], compared with 3 [2, 3] at baseline (*P* < 0.001, **Figure 5G**). Similarly, in the antibody-negative group, the median CASE score at relapse was 3 [2, 4], compared with 5 [3, 6] at baseline (*P* < 0.001, **Figure 5E**). The median mRS score was 2 [2, 2], compared with 3 [2, 4] at baseline (*P* = 0.002, **Figure 5H**). Cross-sectional comparison between the two groups at the time of relapse revealed that antibody-positive patients had higher CASE scores than antibody-negative patients (median 4 [3, 5] vs 3 [2, 4], *P* = 0.017, **Figure 5F**). However, functional disability measured by mRS scores did not statistically differ between the antibody-positive and antibody-negative groups at relapse (median 2 [2, 3] vs 2 [2, 2], *P* = 0.157, **Figure 5I**).

### Structural neuroimaging findings and clinical correlates in autoimmune encephalitis

Inter-rater reliability was assessed separately by antibody serostatus using linearly weighted Cohen κ. In antibody-positive AE, agreement was almost perfect for DCA (κ=0.90, 95% CI 0.85–0.94), mTA (κ=0.92, 95% CI 0.88–0.96), and CHA (κ=0.93, 95% CI 0.85–1.00). Exact agreement was observed in 95.8%, 96.7%, and 99.4% of cases, respectively, with all discrepancies limited to adjacent categories. In antibody-negative AE, agreement remained almost perfect for DCA (κ=0.90, 95% CI 0.84–0.97) and mTA (κ=0.90, 95% CI 0.82–0.98), and was substantial for CHA (κ=0.75, 95% CI 0.59–0.90). Exact CHA agreement was nevertheless 98.1% (104/106), with only adjacent-category discrepancies. Quadratically weighted analyses yielded consistent results.

**Figure 6:**
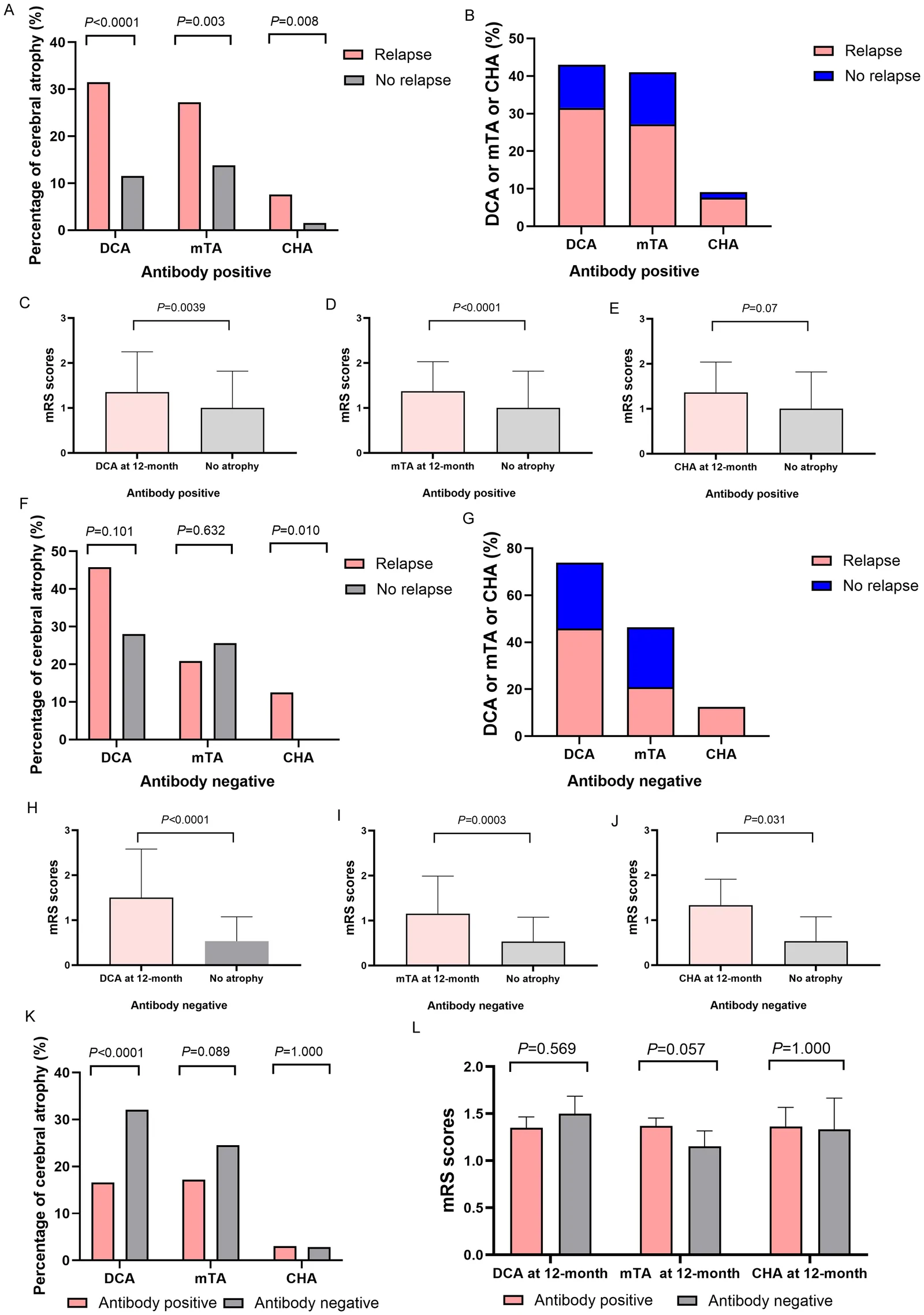
Brain atrophy patterns and clinical correlates in autoimmune encephalitis. **(A)** Distribution of DCA, mTA, and CHA in antibody-positive patients, comparing relapsing and nonrelapsing cases. **(B)** Distribution and frequency of DCA, mTA, and CHA in relapsing and nonrelapsing antibody-positive patients with autoimmune encephalitis. **(C–E)** Functional outcomes (mRS scores) in antibody-positive patients with and without DCA, mTA, or CHA. **(F)** Distribution of DCA, mTA, and CHA in antibody-negative patients, comparing relapsing and nonrelapsing cases. **(G)** Distribution and frequency of DCA, mTA, and CHA in relapsing and nonrelapsing antibody-negative patients with autoimmune encephalitis. **(H–J)** Functional outcomes (mRS scores) in antibody-negative patients with and without DCA, mTA, or CHA. **(K)** Comparison of region-specific brain atrophy patterns (DCA, mTA, and CHA) between antibody-positive and antibody-negative patients with autoimmune encephalitis. **(L)** 12-month mRS scores in patients with brain atrophy, comparing antibody-positive and antibody-negative cohorts. Between-group comparisons were performed using the Mann-Whitney *U* test. Categorical variables were compared using the chi-square test or Fisher exact test, as appropriate. DCA, diffuse cortical atrophy; mTA, medial temporal lobe atrophy; CHA, cerebellar hemisphere atrophy; mRS, modified Rankin scale.

Structural neuroimaging findings differed by antibody serostatus. Among antibody-positive patients, DCA, mTA, and CHA were present in 16.6%, 17.2%, and 3.0%, respectively. Antibody-negative patients had a higher frequency of DCA than antibody-positive patients (32.1% vs 16.6%, *P* < 0.001, **Figure 6K**). The frequencies of mTA (24.5% vs 17.2%, *P* = 0.089) and CHA (2.8% vs 3.0%, *P* = 1.000) did not differ significantly between groups.

Disease relapse was associated with increased frequencies of regional structural injury. Among antibody-positive patients, the relapsing subgroup demonstrated higher atrophy prevalence than the non-relapsing subgroup across all measured regions, including DCA (31.5% vs 11.5%, *P* < 0.001), mTA (27.2% vs 13.8%, *P* = 0.003), and CHA (7.6% vs 1.5%, *P* = 0.008) (**Figure 6A, B**). Within the antibody-negative cohort, relapsing patients exhibited higher CHA frequencies (12.5% vs 0.0%, *P* = 0.010), while differences in DCA (45.8% vs 28.0%, *P* = 0.101) and mTA (20.8% vs 25.6%, *P* = 0.632) were not statistically supported (**Figure 6F, G**). Comparing patients with relapse between the antibody-positive and antibody-negative cohorts, the proportions of atrophy did not differ significantly for DCA (31.5% vs 45.8%, *P* = 0.189), mTA (27.2% vs 20.8%, *P* = 0.528), or CHA (7.6% vs 12.5%, *P* = 0.429).

Across antibody-positive and antibody-negative AE cohorts, combined first-line immunotherapy was not associated with the presence of DCA, mTA, or CHA (all *P*>0.05). In antibody-positive AE, second-line immunotherapy was associated with a higher frequency of DCA (33.3% vs 14.6%, *P* = 0.003), whereas mTA and CHA frequencies did not differ. Long-term immunotherapy was associated with a higher frequency of CHA (7.8% vs 1.8%, *P* = 0.015), with no differences in DCA or mTA. No association between second-line or long-term immunotherapy and regional atrophy was observed in antibody-negative AE (all *P*>0.05). Among patients who relapsed, multiple versus single relapse was not associated with 12-month mRS score or DCA, mTA, or CHA in either serostatus group (all *P*>0.05).

Structural brain atrophy was associated with greater 12-month functional disability. Within the antibody-positive cohort, patients with DCA or mTA recorded higher 12-month mRS scores (median 1 [1, 2]) compared with those without regional atrophy (median 1 [0, 1]) (DCA: *P* = 0.004; mTA: *P* < 0.001; **Figure 6C, D**). CHA in antibody-positive patients was not statistically associated with altered 12-month mRS scores (median 1 [1, 2] vs 1 [0, 1], *P* = 0.070, **Figure 6E**). In the antibody-negative cohort, elevated 12-month mRS scores were recorded in patients with DCA (median 1 [1, 2], *P* < 0.001), mTA (median 1 [1, 1], *P* < 0.001), and CHA (median 1 [1, 2], *P* = 0.031) compared with patients without corresponding regional atrophy (median 1 [0, 1] for all) (**Figure 6H-J**). Comparing patients with specific regional atrophy between the antibody-positive and antibody-negative cohorts, the 12-month mRS scores showed no statistical differences for DCA (median 1 [1, 2] vs 1 [1, 2], *P* = 0.569), mTA (median 1 [1, 2] vs 1 [1, 1], *P* = 0.057), and CHA (median 1 [1, 2] vs 1 [1, 2], *P* = 1.000) (**Figure 6L**).

**Figure 7:**
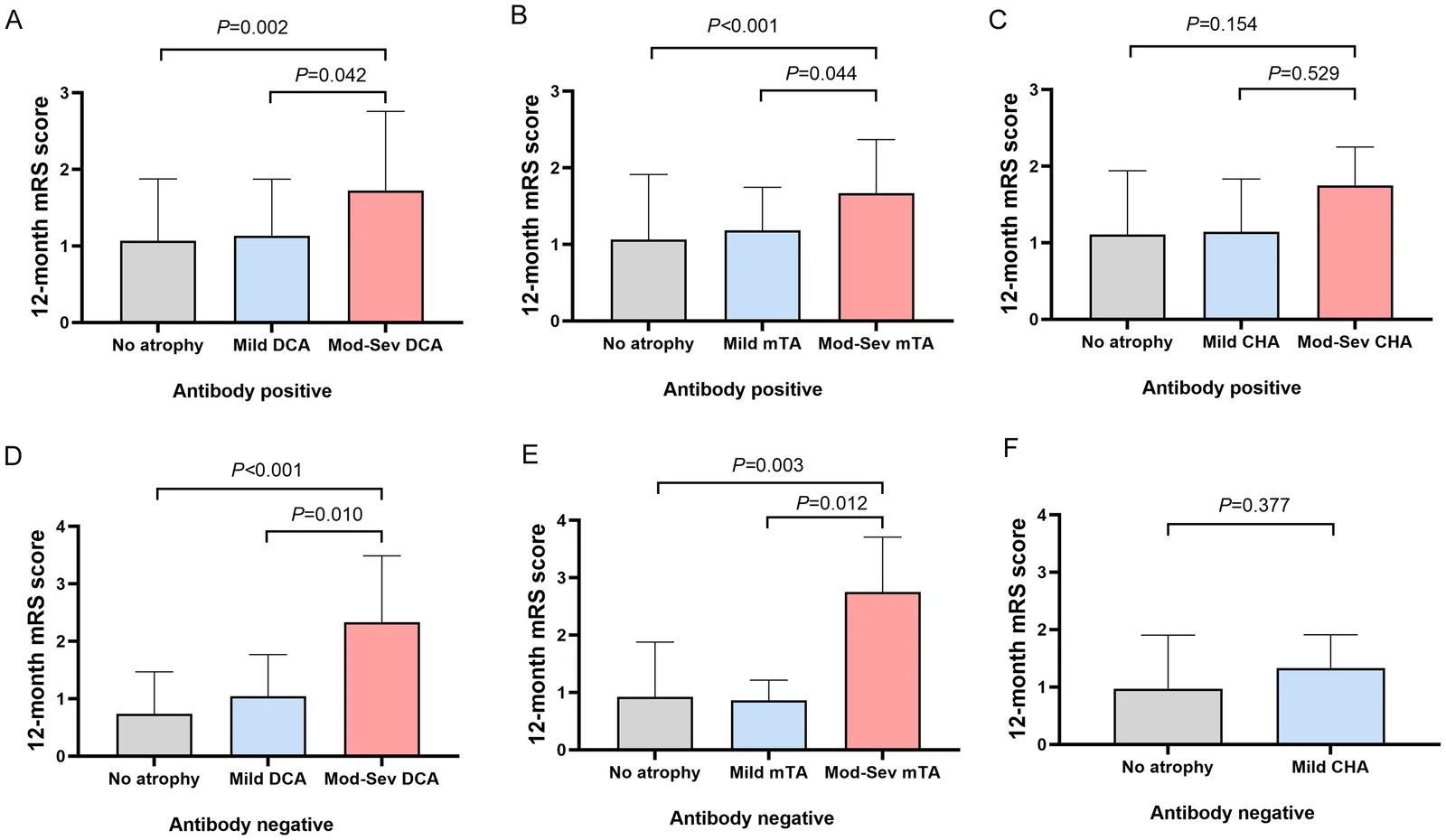
Twelve-month functional disability by regional atrophy severity and antibody serostatus. Twelve-month modified Rankin Scale (mRS) scores are shown according to the severity of diffuse cortical atrophy (DCA), medial temporal atrophy (mTA), and cerebellar hemispheric atrophy (CHA) in antibody-positive AE **(A–C)** and antibody-negative AE **(D–F)**. Atrophy was classified as absent, mild (grade 1), or moderate-to-severe (grades 2–3). Comparisons among multiple groups were performed using the Kruskal–Wallis test followed by Dunn multiple-comparisons test with Bonferroni adjustment; comparisons between 2 groups were performed using the Mann–Whitney *U* test. DCA, diffuse cortical atrophy; mTA, medial temporal lobe atrophy; CHA, cerebellar hemisphere atrophy; mRS, modified Rankin scale.

Further stratification by atrophy severity demonstrated a graded association between atrophy burden and 12-month disability. In antibody-positive AE, moderate-to-severe DCA and mTA were associated with higher 12-month mRS scores than no atrophy (*P* = 0.002 and *P* < 0.001, respectively, **Figure 7A, B**) and mild atrophy (*P* = 0.042 and *P* = 0.044, respectively). Mild DCA or mTA was not significantly different from no atrophy. No severity-dependent association was observed for CHA (**Figure 7C**). In antibody-negative AE, moderate-to-severe DCA and mTA was associated with higher 12-month mRS scores than both no atrophy (*P* < 0.001 and *P* = 0.003, respectively, **Figures 7D, E**) and mild atrophy (*P* = 0.010 and *P* = 0.012, respectively), whereas mild DCA or mTA did not differ from no atrophy. No significant severity-dependent association was observed for CHA (*P* = 0.377, **Figure 7F**).

## Discussion

This large-scale study of 467 patients with autoimmune encephalitis (AE) delineates objective clinical, radiologic, and prognostic distinctions between antibody-positive and antibody-negative subtypes. By integrating clinical phenotypes with longitudinal functional outcomes and structural neuroimaging, our findings provide a data-driven framework for understanding disease heterogeneity and refining prognostic expectations.

Baseline evaluations confirmed distinct pathophysiological signatures between the two cohorts. Antibody-positive AE was characterized by intense compartmentalized neuroinflammation—evidenced by higher cerebrospinal fluid pleocytosis and lower protein levels—and uniquely featured FBDS. Conversely, antibody-negative AE exhibited a broader anatomical burden of acute injury, manifesting as higher frequencies of diffusion restriction across cortical, subcortical, medial temporal, and infratentorial regions. Despite these baseline disparities and the more frequent utilization of intensive immunotherapy in the antibody-positive group, overall mortality rates remained statistically comparable, underscoring the severe natural history of seronegative AE.

Relapse analysis identified divergent, subtype-specific predictive markers across the cohorts. Within the overall antibody-positive cohort, multivariable modeling demonstrated that the presence of FBDS independently predicted disease relapse. However, given that FBDS occurred exclusively in patients with LGI1-antibody encephalitis, a restricted subgroup analysis was essential to disentangle this association. Within the LGI1-seropositive subgroup, multivariable regression revealed that age at onset—rather than the pathognomonic FBDS—emerged as the independent predictor of recurrence. This dissociation highlights an important clinical principle: while FBDS is a pathognomonic clinical hallmark that defines the LGI1 disease, it does not intrinsically capture relapse susceptibility. Notably, older LGI1-positive patients (> 65 years) received first-line combination immunotherapy significantly less frequently than their younger counterparts. This dissociation suggests that the heightened relapse risk observed in the elderly LGI1 demographic is likely mediated, at least in part, by conservative immunotherapeutic management rather than an inherently more aggressive disease course, highlighting a critical area for therapeutic optimization. Conversely, in the antibody-negative cohort, frequent daily seizures were identified as an independent predictor of relapse. Previous studies found that 30.6% of patients experienced relapse and identified refractory status epilepticus as an independent risk factor for relapse in severe antibody-negative autoimmune encephalitis (26).These divergent relapse predictors across antibody strata—FBDS and older age in the seropositive group, and frequent daily seizures in the seronegative group—suggest that the immune mechanisms driving disease reactivation may differ fundamentally between subtypes, warranting further investigation.

Longitudinal tracking demonstrated that while both groups achieved progressive functional improvement following immunotherapy, symptom phenotypes significantly influenced recovery trajectories. Patients with psychiatric-onset antibody-positive AE exhibited persistently higher clinical severity and worse functional outcomes (mRS) at 12 months compared to their antibody-negative counterparts. This trajectory aligns with functional neuroimaging evidence in AE that anterior cingulate hypometabolism independently predicts unfavorable psychiatric recovery, providing a plausible metabolic substrate for persistent deficits (27). In contrast, patients with seizure- or cognition-dominant presentations showed comparable recovery regardless of antibody status. Similarly, age-stratified analyses revealed that while pediatric outcomes were independent of serostatus, elderly antibody-negative patients maintained higher clinical severity (CASE scores) at 12 months, although ultimate functional disability (mRS) did not statistically differ from the antibody-positive elderly cohort. This pattern is consistent with prior evidence indicating that older age at disease onset may be associated with poorer long-term prognosis (26, 28). These findings support a symptom- and age-stratified approach to prognostication over a purely serology-based paradigm.

Evaluation of clinical relapses revealed that recurrences—occurring at a median of 4 to 6 months with no statistical difference between cohorts—presented with attenuated severity compared to the initial episode. This overall clinical attenuation aligns with prior observations and may biologically reflect an absence of progressive epitope spreading during disease reactivation (29, 30). Notably, despite this generalized milder course, antibody-positive patients exhibited significantly higher CASE scores during relapse than the antibody-negative cohort, indicating a more pronounced clinical phenotype upon immune reactivation in seropositive disease.

Our neuroimaging findings identify structural brain atrophy as a clinically relevant correlate of long-term disability across AE serostatus groups. The higher frequency of DCA in antibody-negative AE challenges the assumption that detectable neuronal autoantibodies necessarily denote more extensive structural injury (31). Instead, this finding may reflect a more diffuse pattern of CNS damage in antibody-negative AE, in contrast to the limbic-predominant involvement characteristic of many neuronal surface antibody–mediated syndromes. The biological heterogeneity of antibody-negative AE may encompass blood–brain barrier disruption, microglial activation, and cytotoxic T-cell–mediated neuronal injury, which could collectively contribute to widespread synaptic dysfunction and cortical neurodegeneration (32–35). By comparison, neuronal surface antibody–mediated AE may initially cause antigen-specific and potentially reversible network dysfunction, although persistent inflammation and seizures can result in irreversible structural damage (36–38). Furthermore, disease relapse correlated with accelerated structural injury. Relapsing antibody-positive patients exhibited elevated frequencies of DCA, mTA, and CHA, whereas relapsing antibody-negative patients showed preferential cerebellar vulnerability. Regardless of serostatus, the presence of DCA or mTA was robustly associated with higher 12-month mRS scores. Notably, previous studies have also suggested that the development of cerebellar atrophy may portend an unfavorable prognosis in antibody-negative encephalitis (28). The prognostic impact of these structural changes was comparable between antibody-positive and negative cohorts, indicating that once atrophy occurs, its detrimental effect on functional recovery is independent of the initial immunological trigger.

These findings support a risk-adapted follow-up strategy that integrates serostatus, presenting phenotype, relapse predictors, and structural MRI. Patients with FBDS in the overall antibody-positive cohort, older age at onset in LGI1 encephalitis, or frequent daily seizures in antibody-negative AE warrant closer surveillance for relapse, particularly during the first year, when the median time to relapse was 4–6 months. In these high-risk groups, maintenance immunotherapy should be considered on an individualized basis, with treatment decisions guided by clinical disease activity, relapse history, treatment tolerability, and comorbidities rather than by serostatus alone. Serial MRI assessment of DCA and mTA may provide complementary prognostic information, as moderate-to-severe atrophy was associated with greater 12-month disability in both cohorts. Detection of progressive structural atrophy should prompt reassessment of inflammatory activity, seizure control, and the adequacy of immunotherapeutic management.

This study has several limitations. Its retrospective, multicenter design may have introduced selection bias and between-center heterogeneity in therapeutic strategies. Nonstandardized neuroimaging acquisition protocols may have affected the assessment of structural atrophy, and pre-existing cerebrovascular disease was not systematically excluded and could have confounded atrophy grading. The relatively small antibody-negative cohort, with a limited number of relapse events, constrained the statistical power of multivariable analyses; findings from this subgroup should therefore be interpreted cautiously. Follow-up did not extend beyond 2 years, limiting assessment of longer-term structural and functional trajectories. In addition, the absence of longitudinal peripheral blood and CSF inflammatory biomarker measurements precluded evaluation of dynamic associations between inflammatory activity and neurodegeneration. Prospective multicenter studies incorporating harmonized imaging protocols, standardized treatment strategies, extended follow-up, and serial biomarker assessments are needed to validate these findings.

In conclusion, antibody-positive and antibody-negative AE differed in their clinical characteristics and relapse-associated factors. Across both cohorts, moderate-to-severe DCA and mTA were associated with greater disability at 12 months, whereas no severity-dependent association was observed for CHA. These findings suggest that structural MRI may provide complementary information for prognostic assessment and risk-adapted follow-up in AE. Prospective studies with standardized imaging protocols and longer follow-up are needed to determine whether serial MRI assessment improves clinical decision-making and outcomes.

## Data Availability

The raw data supporting the conclusions of this article will be made available by the authors, without undue reservation.
