# Supplementary material for: Relapse predictors and brain atrophy in antibody-positive and antibody-negative autoimmune encephalitis: a multicenter cohort study
Source: Front Immunol. 2026 Aug 25;17:1879021. doi: 10.3389/fimmu.2026.1879021 (PMC13550121; doi:10.3389/fimmu.2026.1879021)
Supplement: Supplementary file 1 [file SupplementaryFile1.docx]

**Supplementary Figure legends**

Figure S1. Fine–Gray competing-risk regression analysis of relapse in antibody-positive autoimmune encephalitis. Forest plot showing multivariable subdistribution hazard ratios (sHRs) and 95% confidence intervals (CIs) for relapse, with death modeled as a competing event, in 382 patients with antibody-positive autoimmune encephalitis (92 relapses and 21 deaths). Points indicate sHRs and horizontal bars indicate 95% CIs. Orange indicates *P*<0.05; blue indicates *P*≥0.05. FBDS = faciobrachial dystonic seizures; CSF = cerebrospinal fluid.

Figure S2. Fine–Gray competing-risk regression analysis of relapse in antibody-negative autoimmune encephalitis. Forest plot showing multivariable subdistribution hazard ratios (sHRs) and 95% confidence intervals (CIs) for relapse, with death treated as a competing event, in 116 patients with antibody-negative autoimmune encephalitis (24 relapses and 10 deaths). Points indicate sHRs and horizontal bars indicate 95% CIs. Orange indicates *P*<0.05; blue indicates  *P*≥0.05.

**Supplementary Tables**

Table S1 Comparative characteristics of patients with encephalitis: death versus no death cases

| Characteristics | No Death (n=467) | Death (n=31) | *P* |
| --- | --- | --- | --- |
| Female (%) | 194 (41.5) | 14 (45.2) | 0.692 |
| Age at onset (M [P25,P75]), y | 51.0 [27.0, 63.0] | 67.0 [49.0, 72.0] | <0.001 |
| Initial presentation |  | | |
| Neuropsychiatric | 121 (25.9) | 8 (25.8) | 0.990 |
| Seizures | 195 (41.8) | 12 (38.7) | 0.739 |
| Memory dysfunction | 100 (21.4) | 5 (16.1) | 0.485 |
| Other | 51 (10.9) | 6 (19.4) | 0.150 |
| Delayed immunotherapy (%) | 148 (31.7) | 12 (38.7) | 0.418 |
| ≥2ASM | 166 (35.5) | 12 (38.7) | 0.722 |
| ICU admission (%) | 74 (15.8) | 10 (32.3) | 0.018 |
| GCS scores≤13 (%) | 134 (28.7) | 16 (51.6) | 0.007 |
| Any cognitive concerns, n (%) | 408 (87.4) | 26 (83.9) | 0.578 |
| Presence of tumor (%) | 33 ( 7.1) | 10 (32.3) | <0.001 |
| Diffusion restriction (%) |  | | |
| Cortex (%) | 133 (28.5) | 7 (22.6) | 0.479 |
| Subcortex/white matter (%) | 89 (19.1) | 5 (16.1) | 0.687 |
| Medial temporal cortex (%) | 155 (33.2) | 9 (29.0) | 0.633 |
| Infra-tentorium (%) | 23 ( 4.9) | 3 ( 9.7) | 0.215 |
| Video-EEG |  | | |
| Diffuse slow wave | 113 (24.2) | 5 (16.1) | 0.306 |
| Interictal epileptiform discharge | 233 (49.9) | 17 (54.8) | 0.594 |
| Seizures captured | 65 (13.9) | 3 ( 9.7) | 0.786 |
| Initially treated with combined first-line IT, n (%) | 192 (41.1) | 18 (58.1) | 0.064 |
| Initially treated with long-term IT, n (%) | 70 (15.0) | 4 (12.9) | 1.000 |
| Initially treated with second-line IT, n (%) | 29 (6.2) | 2 (6.5) | 1.000 |
| Treated with long-term IT during course, n (%) | 76 (16.3) | 3 (9.7) | 0.450 |
| Treated with second-line IT during course, n (%) | 23 (4.9) | 1 (3.2) | 1.000 |
| combined first-line IT+long-term IT during course | 55 (11.8) | 2 (6.5) | 0.561 |
| combined first-line IT + second-line IT during course | 18 (3.9) | 1 (3.2) | 1.000 |
| Highest seizure frequency, n (%) |  | | |
| Single seizure  Multiple daily/daily  Weekly  Monthly | 26 (5.6) | 1 (3.2) | 1.000 |
|  | 157 (33.6) | 10 (32.3) | 0.877 |
|  | 113 (24.2) | 6 (19.4) | 0.540 |
|  | 47 (10.0) | 5 (16.1) | 0.355 |
| Type of seizure at onset, n (%) |  | | |
| FBDS  Focal seizures  SE | 37 (7.9) | 2 (6.5) | 1.000 |
|  | 339 (72.6) | 17 (54.8) | 0.034 |
|  | 79 (16.9) | 7 (22.6) | 0.419 |
| CSF leucocyte level (10^6^/L) M (P25,P75) | 7 (2,18) | 10 (2,18) | 0.971 |
| CSF protein level (g/L) M (P25,P75) | 0.39 (0.28,0.56) | 0.48 (0.32,0.68) | 0.067 |
| Baseline CASE scores M (P25,P75) | 4 (4,7) | 6 (3,8) | 0.695 |
| Baseline mRS scores M (P25,P75) | 3 (2,3) | 4 (3,4) | 0.002 |

Abbreviations: ASM: antiseizure medication; ICU: Intensive Care Unit; IT: immunotherapy; FBDS: faciobrachial dystonic seizures; SE: status epilepticus; CASE: Clinical Assessment Scale in Autoimmune Encephalitis; mRS, modified Rankin Scale.

Table S2 Comparative characteristics of patients with possible antibody-negative encephalitis and probable antibody-negative encephalitis

| Characteristics | Possible (n=102) | Probable (n=116) | *P* |  |
| --- | --- | --- | --- | --- |
| Female (%) | 37 (31.9) | 46 (45.1) | 0.608 |  |
| Age at onset (range), y | 47.5 (4.0-85.0) | 49.5 (4.0-80.0) | 0.895 |  |
| Initial presentation |  | | |  |
| Neuropsychiatric | 31 (30.4) | 34 (29.3) | 0.862 |  |
| Seizures | 35 (34.3) | 40 (34.5) | 0.979 |  |
| Memory dysfunction | 17 (16.7) | 27 (23.3) | 0.225 |  |
| Impaired consciousness | 6 (5.9) | 5 (4.3) | 0.597 |  |
| Language problem | 2 (2.0) | 4 (3.4) | 0.687 |  |
| Dyskinesia/dystonia | 2 (2.0) | 2 (1.7) | 1.000 |  |
| Gait instability and ataxia | 3 (2.9) | 3 (2.6) | 1.000 |  |
| Other | 6 (5.9) | 1 (0.9) | 0.053 |  |
| Delayed immunotherapy (%) | 31 (30.4) | 36 (31.0) | 0.918 |  |
| ≥2ASM | 23 (22.5) | 40 (34.5) | 0.052 |  |
| ICU admission (%) | 15 (14.7) | 24 (20.7) | 0.250 |  |
| GCS scores≤13 (%) | 36 (35.3) | 42 (36.2) | 0.888 |  |
| Any cognitive concerns, n (%) | 88 (86.3) | 100 (86.2) | 0.988 |  |
| Presence of tumor (%) | 4 (3.9) | 8 (6.9) | 0.337 |  |
| Diffusion restriction (%) |  | | |  |
| Cortex (%) | 1 (1.0) | 62 (53.4) | <0.001 |  |
| Subcortex/white matter (%) | 1 (1.0) | 48 (41.4) | <0.001 |  |
| Medial temporal cortex (%) | 1 (1.0) | 48 (41.4) | <0.001 |  |
| Infra-tentorium (%) | 1 (1.0) | 11 (9.5) | 0.006 |  |
| Video-EEG |  | | |  |
| Diffuse slow wave | 23 (22.5) | 31 (26.7) | 0.476 |  |
| Interictal epileptiform discharge | 48 (47.1) | 56 (48.3) | 0.858 |  |
| Seizures captured | 7 (6.9) | 15 (12.9) | 0.138 |  |
| Initially treated with combined first-line IT, n (%) | 50 (49.0) | 51 (43.9) | 0.455 |  |
| Initially treated with long-term IT, n (%) | 4 (3.9) | 4 (3.4) | 1.000 |  |
| Initially treated with second-line IT, n (%) | 3 (2.9) | 0 (0.0) | 0.101 |  |
| Treated with long-term IT during course, n (%) | 5 (4.9) | 5 (4.3) | 1.000 |  |
| Treated with second-line IT during course, n (%) | 0 (0.0) | 2 (1.7) | 0.500 |  |
| Highest seizure frequency, n (%) |  | | |  |
| Single seizure  Multiple daily/daily  Weekly  Monthly | 2 (2.0) | 5 (4.3) | 0.452 |  |
|  | 28 (27.5) | 30 (25.9) | 0.791 |  |
|  | 22 (21.6) | 28 (24.1) | 0.653 |  |
|  | 15 (14.7) | 12 (10.3) | 0.329 |  |
| Type of seizure at onset, n (%) |  | | |  |
| Focal seizures | 67 (65.7) | 75 (64.7) | 0.873 |  |
| SE | 23 (22.5) | 26 (22.4) | 0.981 |  |
| CSF leucocyte level (10^6^/L) M (P25,P75) | 10.0 (3.0,15.0) | 3.0 (2.0,9.8) | <0.001 |  |
| CSF protein level (g/L) M (P25,P75) | 0.34 (0.28,0.68) | 0.45 (0.30, 0.66) | 0.180 |  |
| CASE scores M (P25,P75) | 5.0 (3.8,6.0) | 5.0 (3.0,6.0) | 0.938 |  |
| Baseline mRS scores M (P25,P75) | 3 (2,4) | 3 (2,4) | 0.607 |  |

Abbreviations: ASM, antiseizure medication; ICU, intensive care unit; EEG, electroencephalography; IT, immunotherapy; FBDS, faciobrachial dystonic seizures; SE, status epilepticus; CASE, Clinical Assessment Scale in Autoimmune Encephalitis; mRS, modified Rankin Scale.

Table S3 Comparative characteristics of older patients (≥65 years) with LGI 1 antibody-positive encephalitis: relapsing versus non-relapsing cases

| Characteristics | Relapse (n=20) | No relapse (n=27) | *P* |
| --- | --- | --- | --- |
| Female (%) | 8 (40.0) | 12 (44.4) | 0.761 |
| Age at onset (range), y | 69 (65-81) | 67 (65-87) | 0.166 |
| Initial presentation |  | | |
| Neuropsychiatric | 4 (20.0) | 1 (3.7) | 0.148 |
| Seizures | 10 (50.0) | 19 (70.4) | 0.155 |
| Memory dysfunction | 4 (20.0) | 6 (22.2) | 1.000 |
| Other | 2 (10.0) | 1 (3.7) | 0.567 |
| Delayed immunotherapy (%) | 8 (40.0) | 12 (44.4) | 0.761 |
| Poor adherence (%) | 3 (15.0) | 2 (7.4) | 0.638 |
| ≥2ASM | 6 (30.0) | 12 (44.4) | 0.314 |
| ICU admission (%) | 1 (5.0) | 1 (3.7) | 1.000 |
| GCS scores≤13 (%) | 4 (20.0) | 8 (29.6) | 0.454 |
| Any cognitive concerns, n (%) | 15 (75.0) | 23 (85.2) | 1.000 |
| Presence of tumor (%) | 3 (15.0) | 1 (3.7) | 0.298 |
| Diffusion restriction (%) |  | | |
| Cortex (%) | 2 (10.0) | 3 (11.1) | 1.000 |
| Subcortex/white matter (%) | 0 (0.0) | 1 (3.7) | 1.000 |
| Medial temporal cortex (%) | 4 (20.0) | 11 (40.7) | 0.132 |
| Infra-tentorium (%) | 0 (0.0) | 3 (11.1) | 0.251 |
| Video-EEG |  | | |
| Diffuse slow wave | 6 (30.0) | 4 (14.8) | 0.286 |
| Interictal epileptiform discharge | 12 (60.0) | 18 (66.7) | 0.638 |
| Seizures captured | 1 (5.0) | 2 (7.4) | 1.000 |
| Initially treated with combined first-line IT, n (%) | 7 (35.0) | 14 (51.9) | 0.251 |
| Initially treated with long-term IT, n (%) | 3 (15.0) | 7 (25.9) | 0.481 |
| Treated with long-term IT during course, n (%) | 4 (20.0) | 7 (25.9) | 0.737 |
| Treated with second-line IT during course, n (%) | 0 (0.0) | 1 (3.7) | 1.000 |
| combined first-line IT+long-term IT during course | 3 (15.0) | 4 (14.8) | 1.000 |
| combined first-line IT + second-line IT during course | 0 (0.0) | 1 (3.7) | 1.000 |
| Highest seizure frequency, n (%) |  | | |
| Single seizure  Multiple daily/daily  Weekly  Monthly | 1 (5.0) | 3 (11.1) | 0.626 |
|  | 9 (45.0) | 14 (51.9) | 0.642 |
|  | 5 (25.0) | 7 (25.9) | 0.943 |
|  | 2 (10.0) | 2 (7.4) | 1.000 |
| Type of seizure at onset, n (%) |  | | |
| FBDS  Focal seizures  SE | 7 (35.0) | 11 (40.7) | 0.689 |
|  | 17 (85.0) | 25 (92.6) | 0.638 |
|  | 2 (10.0) | 2 (7.4) | 1.000 |
| CSF leucocyte level (10^6^/L) M (P25,P75) | 3.0 (2.0,10.0) | 3.0 (2.0,10.5) | 0.455 |
| CSF protein level (g/L) M (P25,P75) | 0.43 (0.31,0.56) | 0.30 (0.24,0.46) | 0.109 |
| Baseline CASE scores M (P25,P75) | 5 (4,6) | 5 (4,7) | 0.979 |
| Baseline mRS scores M (P25,P75) | 3 (2,3) | 3 (2,4) | 0.563 |

Abbreviations: ASM: antiseizure medication; ICU: Intensive Care Unit; IT: immunotherapy; FBDS: faciobrachial dystonic seizures; SE: status epilepticus; CASE: Clinical Assessment Scale in Autoimmune Encephalitis; mRS, modified Rankin Scale.
